# Endovascular treatment for Behçet’s disease: a case report

**DOI:** 10.1590/1677-5449.180121

**Published:** 2019-06-08

**Authors:** Sergio Quilici Belczak, Ingredy Tavares da Silva, Gustavo Garcia Marques, Letícia Flores Copetti, Vanessa Stefaniak, Gustavo Gomes Quintas, Kaique Bernardo Uchimura

**Affiliations:** 1 Centro Universitário São Camilo, São Paulo, SP, Brasil.; 2 Instituto de Aprimoramento e Pesquisa em Angiorradiologia e Cirurgia Endovascular – IAPACE, São Paulo, SP, Brasil.

**Keywords:** Behçet syndrome, endovascular procedures, stents, aneurysm

## Abstract

Behçet’s disease (BD) is a multisystemic vasculitis of unknown etiology. Cardiovascular involvement has been thoroughly described in the literature and the major cause of death in BD is secondary to aneurysm complications. In this case report, a patient with BD presented with a recurrent abdominal aortic aneurysm, which was corrected using a custom-made endoprosthesis. The optimal treatment for patients with BD remains highly controversial and challenging because of technical difficulties and frequent recurrence. Endovascular intervention seems to be a feasible alternative with considerably less morbidity than conventional surgery.

## INTRODUCTION

Behçet’s disease (BD) is a multisytemic vasculitis first described in 1937 by Hulusi Behçet, characterized by a triad comprising recurrent aphthous stomatitis, genital ulceration, and refractory uveitis.^1^ It is now known that it involves multiple systems, including mucocutaneous, ocular, articular, neurological, cardiovascular, gastrointestinal, and respiratory systems.^2^

The disease is most prevalent among men aged 20 to 40 years and is generally seen in Mediterranean countries, the Middle East, and Japan, but it has been described worldwide.^2^ Diagnosis is essentially clinical and there is no specific diagnostic test.^3^^,^ 4 There is an international criterion for diagnosis/classification of BD, based on a scoring system that attributes two points each to ocular lesions and genital ulcers and one point to each of the following items: oral apthous ulcers; cutaneous manifestations; vascular manifestations; and a positive pathergy test. Three or more points are needed to define a patient as a case of BD.^5^

Studies are still being conducted in attempts to uncover the pathogenesis of this disease, which remains obscure and the subject of speculation.^1^^,^ 6 Notwithstanding, a relationship with the HLA-B5 gene has been demonstrated in many patients.^7^ Pathophysiology appears to involve vasculitis of the vasa vasorum with adventitial fibrosis, destruction of elastic and muscle fibers of the tunica media and thickening of the tunica intima, which would explain the presence of thromboses in vessels of small diameter and dilation or rupture of larger vessels.^8^^,^ 9 Prognosis is variable, depending on the principal manifestations in each patient. Men generally have more severe presentations and, consequently, worse prognosis.^1^^,^ 7

The principal cause of death in BD is secondary to vascular complications, and the most common is aneurysmal rupture.^6^^,^ 10 ^,^ 11 Early diagnosis and treatment are therefore indispensable to reduce mortality.

## CASE DESCRIPTION

**Figure 1:**
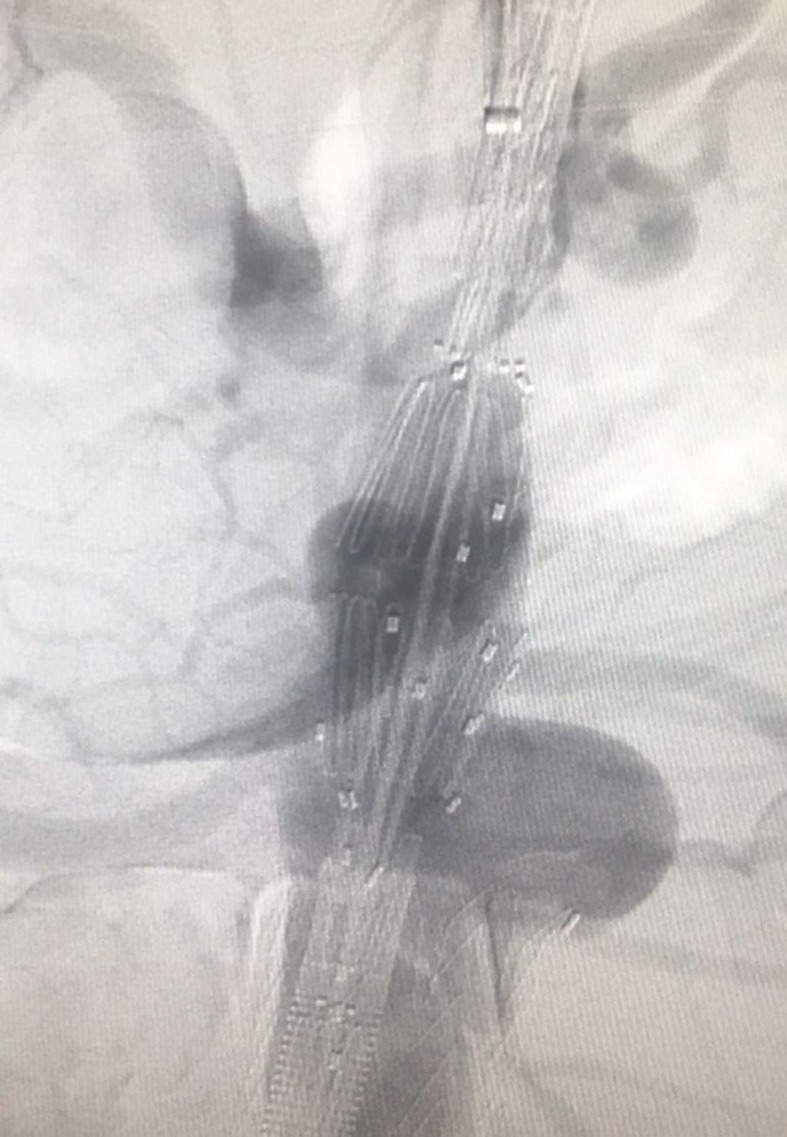
Initial angiography before placement of the second endoprosthesis.

A 38 year-old, male patient with BD diagnosed 2 years previously, with no typical current symptom of this disease, presented with signs of rupture of an infrarenal abdominal aortic aneurysm. He therefore underwent endovascular surgical treatment. After placement of a stent (Figure 1), during the immediate postoperative period, occlusion of the left iliac artery was detected, with signs of ischemia spreading to involve the entire limb. Immediate management was conducted using a Fogarty catheter, followed by placement of a bovine pericardium patch, with compensation of the limb. There was occlusion at this site, but since perfusion of the limb was maintained and the limb was painless, with no claudication or signs of ischemia, the decision was taken to manage the patient with conservative monitoring. As part of postoperative follow-up, serial computed tomography examinations were performed, initially three-monthly and then six-monthly. Approximately 2 years after the aneurysm just described, an examination showed a possible area of de novo rupture, in the aorta, with bleeding into the retroperitoneal space, in the juxtarenal area.

During careful analysis of the case, consideration was given to the fact that the patient had been stable for some weeks after the examination and that he had a delicate pathological history, in view of the vascular complications involving the aortic segment. A further endovascular intervention was therefore planned, with the intention of achieving a definitive repair, using a custom endoprosthesis, i.e., made-to-measure by COOK^®^ (Cook Medical, Limerick, Ireland). This endoprosthesis arrived in São Paulo, SP, Brazil after approximately 100 days.

**Figure 2:**
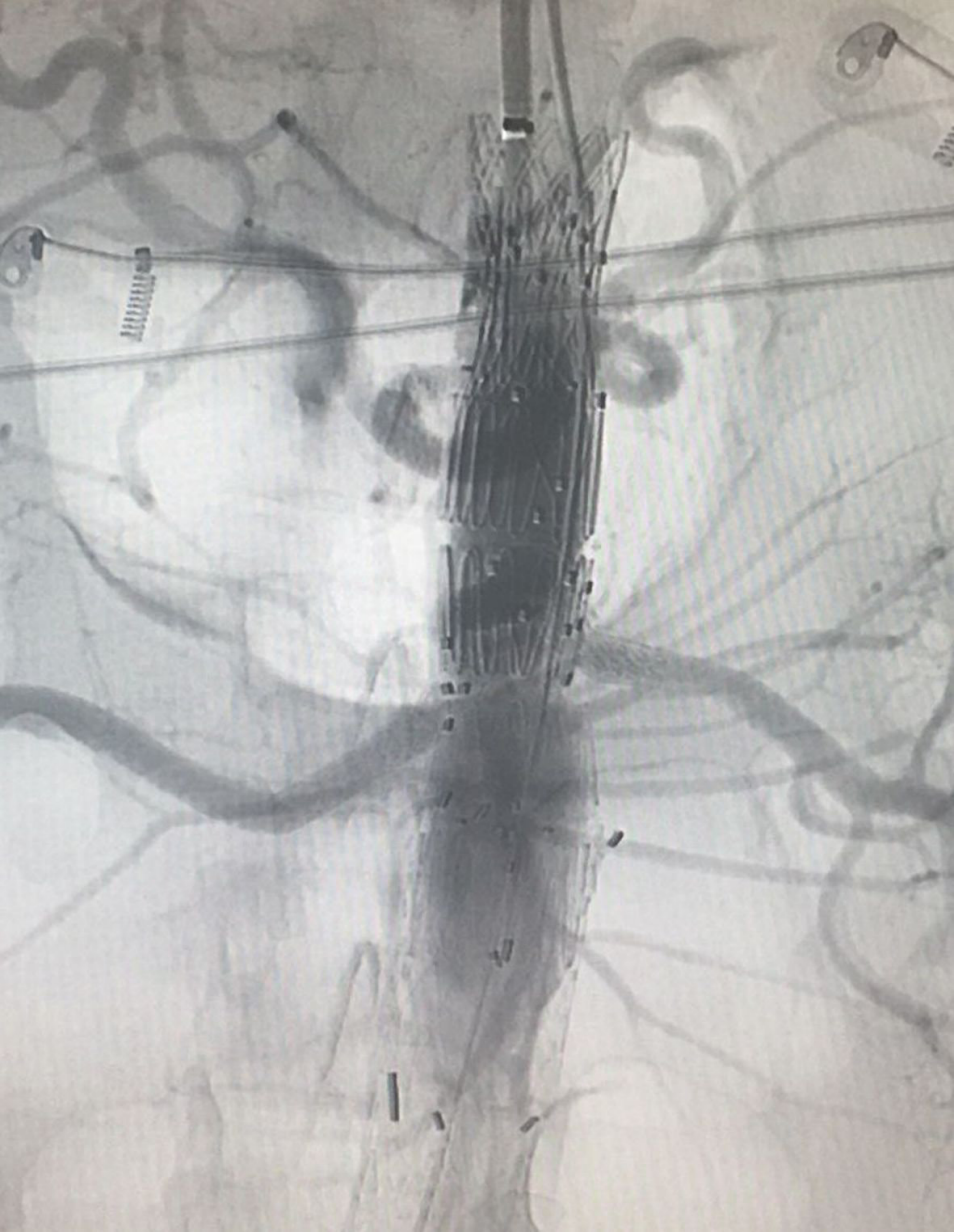
Control angiography after deployment of the second endoprosthesis and the covered stents.

**Figure 3:**
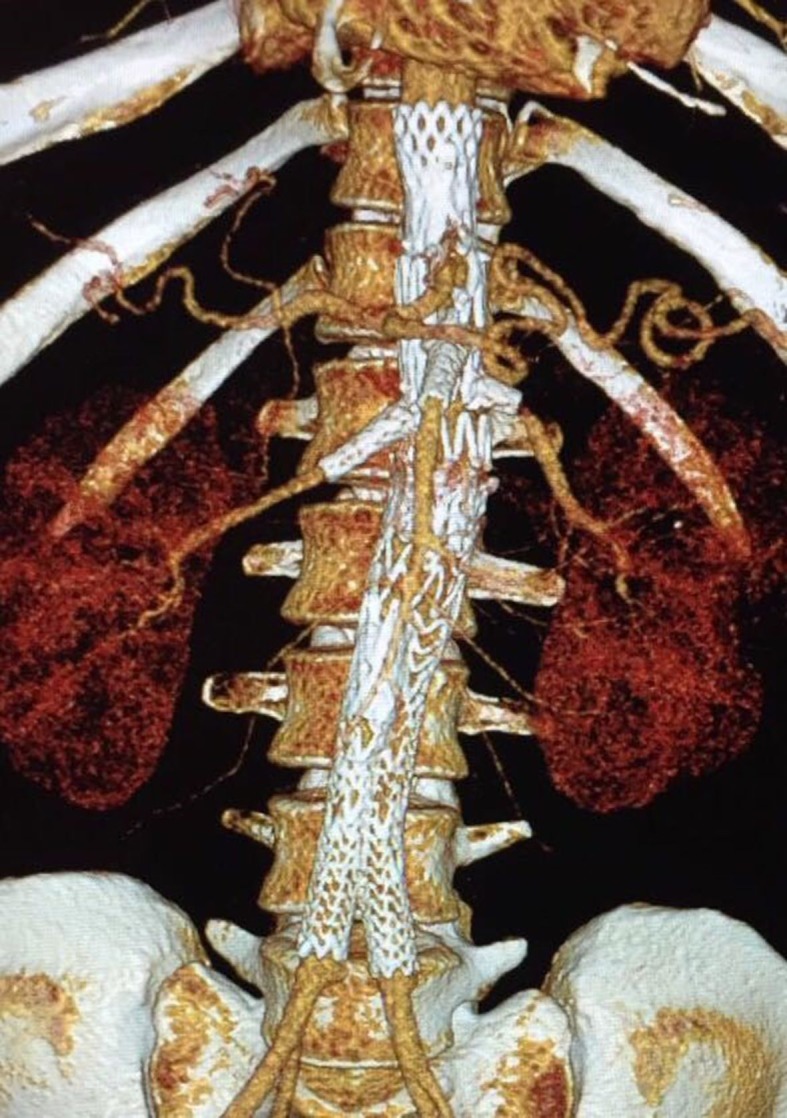
Three-dimensional reconstruction of control angiotomography at 6-month follow-up.

During the surgical procedure, arterial access was obtained via the right femoral and right brachial arteries. The custom endoprosthesis was then deployed. This item had a “scallop” in the celiac trunk designed to avoid suppressing the flow to subsequent arteries and fenestrations for the superior mesenteric and renal arteries, giving rise to a further three covered stents (Advanta^®^, Atrium Medical Corporation, Merrimack, United States). The procedure was accomplished with no intercurrent complications, as shown by control angiography conducted after release of the implants (Figure 2). The patient attended for follow-up with the rheumatology team and was put on corticoid therapy at immunosuppressant doses in combination with cyclophosphamide. He has been in follow-up for more than 12 months and no further complications have been detected, as shown by control reconstruction tomography (Figure 3).

## DISCUSSION

Behçet’s disease is characterized by chronic systemic vasculitis involving the mucocutaneous, occular, articular, neurological, cardiovascular, gastrointestinal, and respiratory systems.^2^^,^ 7 ^,^ 12 ^,^ 13 It most often occurs in young male adults between the third and fourth decades of life.^2^^,^ 6 ^,^ 9 ^,^ 14 The case described here fits the most common epidemiological profile found in the literature. It is now recognized that the vascular injuries of BD are more common among male patients.^10^^,^ 13

In the literature, rates of vascular involvement are variable, affecting from 7 to 29% of patients with BD.^2^^,^ 4 ^,^ 8 ^,^ 11 ^,^ 15 ^,^ 16 The disease can involve both the venous system and the arterial system, causing thromboses venous/thrombophlebitis and varicose veins in around 88% of patients with vascular injuries. Arterial involvement, including occlusions/stenoses, aneurysms, and pseudoaneurysms, are responsible for approximately 12% of cases.^1^^-^ 4 ^,^ 6 ^,^ 7 ^,^ 12 ^,^ 16 ^,^ 17 Although less frequent, arterial injuries, such as aneurysms, are the major cause of death.^2^^,^ 15 ^,^ 18 The abdominal aorta is the most commonly affected site, followed by the femoral and pulmonary arteries.^3^^,^ 4 ^,^ 6 ^-^ 8 ^,^ 10 ^,^ 18

The origin of BD it is not yet clear,^2^^,^ 7 ^,^ 15 but it is believed that an autoimmune phenomenon takes place and triggers an inflammatory process. This causes degeneration of the vasa vasorum, thickening of the tunica intima, and destruction of the elastic fibers of the walls of vessels.^2^^,^ 3 ^,^ 6 ^,^ 8 ^-^ 10 ^,^ 12 ^,^ 14 ^,^ 16 ^,^ 19 In turn, the thrombotic phenomena can be explained by reduced fibrinolytic activity with increased platelet aggregation, caused by the inflammatory state and the reduction in prostacyclin production.^13^

Administration of corticosteroids is therefore recommended before and after surgical repair of these aneurysms. In some cases, immunosuppressants are used to provoke a remission of the disease, reducing inflammation of the vessels and the risk of postoperative complications.^2^^,^ 3 ^,^ 4 ^,^ 6 ^,^ 7 ^,^ 12 ^-^ 15 ^,^ 17 It is known that aneurysms caused by BD exhibit a vigorous inflammatory process in the artery walls, expand rapidly, and have an elevated risk of rupture that is not correlated with their size and, because of this, must be treated whenever possible.^10^^,^ 11 Treatment of the vascular injuries seen in these patients is a major challenge for vascular surgeons, because of the technical difficulties involved and the high rates of complications, such as formation of pseudoaneurysms at anastomosis sites, which occur in 30 to 50% of patients with BD.^2^^,^ 4 ^,^ 10 ^,^ 12 ^,^ 16

For a long time, open surgical repair was the standard treatment for aneurysms in patients with BD. However, these procedures tend to produce results that are very often unsatisfactory, in terms of the considerable morbidity and mortality and the high rates of recurrence.^9^^,^ 15 ^,^ 16 This was demonstrated in a study by Park et al.,^20^ who assessed the results of conventional surgery in 37 patients with BD and found a 35.9% incidence of complications.

In this context, endovascular treatment is a promising option that offers reduced surgery time and shorter hospital stays. It also causes less blood loss and has lower mortality rates. The literature indicates that the endovascular technique is effective (success rates exceed 90%) and safe, with lower mortality (rates range from 0.6 to 3.5%), and fewer complications during the postoperative period (incidence of around 19%), resulting in more rapid recovery. For these reasons it should be the option of choice in the majority of cases, particularly when surgical risk is high.^5^^,^ 7 ^,^ 8 ^,^ 10 ^,^ 15

In a study of 912 patients with BD, 20 aneurysms in 16 patients were treated with endovascular techniques. In counterpoint, eight aneurysms in seven patients were treated with repair by conventional surgery. It was observed that endovascular procedures were successful in all lesions. Mean follow-up was 47 months, during which there were four complications in three of the 16 patients (18.8%) treated with the minimally invasive technique. Among the seven patients treated with open surgery, there were three (42.9%) complications during follow-up, one of which involved a death.^10^

One of the possible complications of endovascular treatment is endoleaks.^10^ In the case of our patient, he had already undergone endovascular treatment for an infrarenal abdominal aortic aneurysm and then developed an aneurysm above the prosthesis implanted previously, in the topography of the renal artery. There were no complications such as thromboses or endoleaks after correction of the second aneurysm using the prosthesis designed specifically for this patient, according to angiotomography, which covered an area larger than the area involved, reaching the celiac trunk.

In order to avoid the complications already mentioned, in addition to using corticosteroids/immunosuppressants and heparin (before placement), it is recommended that, if possible, anastomoses should be constructed in segments that are macroscopically free from inflammation, covering a larger area.^4^^,^ 9 ^,^ 16 In a study with nine aneurysms in seven patients, stents with diameters around 10 to 15% larger than the aorta were placed proximal and distal of the lesion, and length was sized to cover an additional 2 cm to leave the border further from the aneurysm. In one case, an aortic stent was designed on the basis of analysis of computed tomography. All of these aneurysms were repaired successfully.^8^

The medical industry offers a wide range of types of stents. However, in these cases, the ideal option is to construct made-to-measure prostheses, after detailed analysis of imaging exams, customizing them for each patient,^8^^,^ 19 ^,^ 21 as was done in the case described here. In patients with BD, arterial aneurysms expand rapidly, increasing the risk of rupture. Repair is therefore mandatory, although the ideal treatment is still controversial and challenging, bearing in mind the technical difficulties and frequent relapses. Endovascular intervention is a feasible option that should be employed, since conventional surgery morbidity and mortality rates remain high. It should be emphasized that, irrespective of the treatment chosen to repair vascular injuries, it is necessary to control inflammatory activity by immunosuppression, before and after the procedure.
