# Supplementary material for: Endovascular treatment for Behçet’s disease: a case report
Source: J Vasc Bras. 2019 Jun 8;18:e20180121. doi: 10.1590/1677-5449.180121 (PMC6636910; doi:10.1590/1677-5449.180121)
Supplement: Translation

# Translation: Tratamento endovascular da doença de Behçet: relato de caso

**Authors:** Sergio Quilici Belczak, Ingredy Tavares da Silva, Gustavo Garcia Marques, Letícia Flores Copetti, Vanessa Stefaniak, Gustavo Gomes Quintas, Kaique Bernardo Uchimura

**Affiliations:** 1 Centro Universitário São Camilo, São Paulo, SP, Brasil.; 2 Instituto de Aprimoramento e Pesquisa em Angiorradiologia e Cirurgia Endovascular – IAPACE, São Paulo, SP, Brasil.

**Keywords:** Síndrome de Behçet, procedimentos endovasculares, stents, aneurisma

## Abstract

A doença de Behçet (DB) é uma vasculite multissistêmica de etiologia desconhecida. O acometimento cardiovascular é bem descrito na literatura, sendo que a principal causa de morte na DB é secundária a complicações aneurismáticas. Relata-se aqui o caso de um paciente portador de DB com aneurisma de aorta abdominal reincidente, o qual foi corrigido por meio de endoprótese customizada. O tratamento ideal para portadores de DB ainda é bastante controverso e desafiador, tendo em vista as dificuldades técnicas e as recidivas frequentes. A intervenção endovascular apresenta-se como uma alternativa viável e consideravelmente menos mórbida do que a cirurgia convencional.

## INTRODUÇÃO

A doença de Behçet (DB) é uma vasculite multissistêmica descrita em 1937, por Hulusi Behçet, e caracterizada pela tríade: estomatite aftosa recorrente, ulceração genital e uveíte recidivante^1^. Atualmente, sabe-se que ela acomete múltiplos sistemas, incluindo envolvimento mucocutâneo, ocular, articular, neurológico, cardiovascular, gastrointestinal e respiratório^2^.

A doença é mais prevalente em homens entre 20 e 40 anos e geralmente observada em países mediterrâneos, no Oriente Médio e no Japão, mas com descrição em todo o mundo^2^. O diagnóstico é essencialmente clínico, não havendo um exame diagnóstico específico^3^^,^ 4. Pode-se utilizar o critério internacional para diagnóstico/classificação da DB, composto por um sistema de pontuação que atribui dois pontos para lesões oculares e úlceras genitais cada, e um ponto para cada um dos seguintes itens: aftose oral; manifestações cutâneas; manifestações vasculares; e teste de patergia positiva. Três ou mais pontos são necessários para considerar o paciente um caso de DB^5^.

Ainda estão sendo realizados estudos para elucidar a patogênese da doença, que continua obscura e especulativa^1^^,^ 6. No entanto, foi demonstrada uma relação com o gene HLA-B5 em muitos pacientes^7^. A fisiopatologia parece envolver vasculite de *vasa vasorum* com fibrose adventícia, destruição das fibras elásticas e musculares da camada média e espessamento da camada íntima, o que explicaria a presença de trombose em vasos de pequeno diâmetro e dilatação ou ruptura em vasos maiores^8^^,^ 9. O prognóstico da DB é variável, dependendo dos principais acometimentos do paciente. Homens geralmente têm apresentação mais grave e, portanto, pior prognóstico^1^^,^ 7.

A principal causa de morte na DB é secundária a complicações vasculares, sendo a principal delas o aneurisma roto^6^^,^ 10 ^,^ 11. Assim, é imprescindível realizar o diagnóstico e o tratamento precocemente para reduzir a mortalidade.

## DESCRIÇÃO DO CASO

Paciente de 38 anos, do sexo masculino, portador de DB diagnosticada há 2 anos, sem nenhum sintoma típico atual desta doença, apresentou sinais de ruptura de um aneurisma de aorta abdominal infrarrenal. Por isso, foi submetido a tratamento cirúrgico endovascular. Ao colocar uma endoprótese (Figura 1), evoluiu no pós-operatório imediato com oclusão da artéria ilíaca esquerda, com sinais de isquemia, que irradiou para todo o membro. A abordagem imediata foi realizada com cateter Fogarty e, posteriormente, com *patch* de pericárdio bovino, e o membro foi compensado. Houve oclusão desse sítio, mas, frente à conservação da perfusão do membro, o qual se manteve indolor, sem claudicação ou sinais de isquemia, optou-se por conduta expectante. No seguimento pós-operatório, foram realizadas tomografias computadorizadas seriadas, inicialmente com frequência trimestral e, após, semestral. Aproximadamente 2 anos depois do aneurisma citado, um exame evidenciou nova área de possível ruptura contida na aorta com sangramento para o retroperitônio, na porção justarrenal.

**Figura 1:** Angiografia inicial antes do implante da segunda endoprótese.

Após criteriosa análise do caso, considerou-se que o paciente estava estável há semanas desde o exame e que apresentava delicado histórico patológico devido a complicações vasculares no segmento aórtico. Logo, foi indicado novo tratamento endovascular, visando correção definitiva, dessa vez com uma endoprótese customizada, isto é, feita sob medida pela COOK^®^ (Cook Medical, Limerick, Irlanda). A endoprótese demorou aproximadamente 100 dias para chegar a São Paulo (SP), Brasil.

Durante o procedimento cirúrgico, foi obtido acesso arterial via femoral direita e braquial direita. Foi implantada a endoprótese sob medida, que dispunha de um *scallop* no tronco celíaco para não suprimir a irrigação para as artérias subsequentes e de fenestras para as artérias mesentérica superior e renais, dispondo assim de mais três stents revestidos (Advanta^®^, Atrium Medical Corporation, Merrimack, EUA). O procedimento ocorreu sem intercorrências evidenciadas pela angiografia de controle após a liberação dos implantes (Figura 2). Reitera-se que o paciente faz acompanhamento conjunto com equipe de reumatologia e fez uso de corticoterapia em doses imunossupressoras associada a ciclofosfamida. O paciente mantém acompanhamento há mais de 12 meses e não apresentou novas complicações, conforme demonstra a reconstrução tomográfica de controle (Figura 3).

**Figura 2:** Angiografia de controle após o implante da segunda endoprótese e dos stents revestidos.

**Figura 3:** Reconstrução tridimensional da angiotomografia de controle do paciente após 6 meses de evolução.

## DISCUSSÃO

A DB é caracterizada por uma vasculite sistêmica crônica que repercute nos sistemas mucocutâneo, ocular, articular, neurológico, cardiovascular, gastrointestinal e respiratório^2^^,^ 7 ^,^ 12 ^,^ 13. Aparece mais comumente em adultos jovens do sexo masculino, entre a terceira e a quarta décadas de vida^2^^,^ 6 ^,^ 9 ^,^ 14. O caso relatado apresenta o perfil epidemiológico mais encontrado na literatura. Atualmente, reconhece-se que as lesões vasculares são mais comuns nos pacientes do sexo masculino^10^^,^ 13.

Na literatura, tem-se observado uma taxa de acometimento vascular variável, afetando entre 7 e 29% dos pacientes com DB^2^^,^ 4 ^,^ 8 ^,^ 11 ^,^ 15 ^,^ 16. A doença pode envolver tanto o sistema venoso quanto o arterial, ocasionando tromboses venosas/tromboflebites e varizes em cerca de 88% dos pacientes com lesões vasculares. Já os acometimentos arteriais, como oclusões/estenoses, aneurismas e pseudoaneurismas, são responsáveis por aproximadamente 12% dos casos^1^^-^ 4 ^,^ 6 ^,^ 7 ^,^ 12 ^,^ 16 ^,^ 17. Embora menos frequentes, as lesões arteriais, como aneurismas, são as principais causas de morte^2^^,^ 15 ^,^ 18. O local mais acometido é a aorta abdominal, seguida das artérias femoral e pulmonar^3^^,^ 4 ^,^ 6 ^-^ 8 ^,^ 10 ^,^ 18.

A origem da DB ainda não está clara^2^^,^ 7 ^,^ 15, mas acredita-se que um fenômeno autoimune ocorra e desencadeie um processo inflamatório. Este leva a uma degeneração de *vasa vasorum*, espessamento da camada íntima e destruição das fibras elásticas das paredes dos vasos^2^^,^ 3 ^,^ 6 ^,^ 8 ^-^ 10 ^,^ 12 ^,^ 14 ^,^ 16 ^,^ 19. Já os fenômenos trombóticos são explicados pela ação fibrinolítica reduzida com agregação plaquetária aumentada, devido ao estado inflamatório e à diminuição da produção de prostaciclinas^13^.

Portanto, é preconizado o uso de corticoides antes e após o reparo cirúrgico dos aneurismas. Em alguns casos, imunossupressores são utilizados para induzir a remissão da doença, diminuindo a inflamação nos vasos e o risco de complicações pós-operatórias^2^^,^ 3 ^,^ 4 ^,^ 6 ^,^ 7 ^,^ 12 ^-^ 15 ^,^ 17. Sabe-se que os aneurismas oriundos da DB apresentam elevado processo inflamatório nas paredes arteriais, têm rápida expansão e elevado risco de ruptura não correlacionado ao tamanho e, por isso, devem ser tratados sempre que possível^10^^,^ 11. O tratamento das lesões vasculares nesses pacientes é um grande desafio para o cirurgião vascular, devido às dificuldades técnicas associadas e ao grande índice de complicações, como a formação de pseudoaneurismas em locais anastomóticos, que acometem de 30 a 50% dos pacientes com DB^2^^,^ 4 ^,^ 10 ^,^ 12 ^,^ 16.

O reparo cirúrgico aberto foi, por muito tempo, a terapia padrão para correção de aneurismas em pacientes com DB. No entanto, o procedimento costuma apresentar resultados muitas vezes insatisfatórios, devido à importante morbimortalidade relacionada e às altas taxas de recorrência^9^^,^ 15 ^,^ 16. Isso foi evidenciado no estudo de Park et al.^20^, que avaliaram os resultados da cirurgia convencional em 37 pacientes com DB e encontraram uma incidência de complicações de 35,9%.

Nesse contexto, o tratamento endovascular é uma terapia promissora que resulta na redução do tempo cirúrgico e do tempo de internação hospitalar. Além disso, causa menor perda sanguínea e apresenta taxas da mortalidade reduzidas. A literatura aponta que a técnica endovascular é efetiva (taxas de sucesso superiores a 90%) e segura, apresentando menor mortalidade (taxas de 0,6% a 3,5%) e menos complicações no pós-operatório (incidência de cerca de 19%), levando a uma recuperação mais rápida. Por isso, deve ser a abordagem de escolha na maioria dos casos, principalmente quando o risco cirúrgico é alto^5^^,^ 7 ^,^ 8 ^,^ 10 ^,^ 15.

Em um estudo envolvendo 912 pacientes com DB, 20 aneurismas presentes em 16 pacientes foram tratados com terapia endovascular. Em contrapartida, oito aneurismas presentes em sete pacientes foram tratados por correção mediante cirurgia convencional. Verificou-se que o procedimento endovascular foi bem-sucedido em todas as lesões. O seguimento médio foi de 47 meses, durante o qual ocorreram quatro complicações em três dos 16 pacientes (18,8%) tratados com técnica minimamente invasiva. Nos sete pacientes submetidos a cirurgia aberta, três (42,9%) complicações ocorreram no seguimento, com uma morte relacionada^10^.

Entre as complicações da terapia endovascular estão os *endoleaks* 10. No caso do nosso paciente, ele já havia sido submetido a tratamento endovascular de aneurisma de aorta abdominal infrarrenal e evoluiu com um aneurisma acima da prótese que já possuía, na topografia da artéria renal. Não existiram complicações como tromboses ou *endoleaks* após a correção do segundo aneurisma com a prótese individualmente projetada, de acordo com a angiotomografia, que cobriu uma área maior do que a área afetada, chegando até o tronco celíaco.

Para evitar as complicações já citadas, além do uso de corticoides/imunossupressores e heparina (antes da inserção), sugere-se a tentativa de realizar anastomoses, se possível, em segmentos macroscopicamente isentos de inflamação, cobrindo uma área maior^4^^,^ 9 ^,^ 16. Em um estudo com nove aneurismas em sete pacientes, aplicaram-se stents com diâmetro cerca de 10 a 15% maior que o da aorta, proximal e distal à lesão, e comprimento cobrindo 2 cm a mais para distanciar a margem do aneurisma. Em um caso, o stent aórtico foi projetado com base na análise da tomografia computadorizada. Todos foram corrigidos com sucesso^8^.

A indústria disponibiliza diversos tipos de stents. Porém, nesses casos, o ideal é que a confecção das próteses seja feita sob medida, após análise detalhada dos exames de imagem, tornando-se personalizada para cada paciente^8^^,^ 19 ^,^ 21, assim como se procedeu no caso relatado. Em pacientes portadores de DB, os aneurismas arteriais evoluem com rápida expansão e aumentado risco de ruptura. Logo, exigem correção, embora o tratamento ideal ainda seja controverso e desafiador, tendo em vista as dificuldades técnicas e as recidivas frequentes. A intervenção endovascular é uma alternativa viável que deve ser aplicada, uma vez que a morbimortalidade da cirurgia convencional segue elevada. Reitera-se que, independentemente da terapia aplicada para corrigir as lesões vasculares, é necessário realizar o controle da atividade inflamatória por meio de imunossupressão, antes e após o procedimento.
